# An Shen Ding Zhi Ling Alleviates Symptoms of Attention Deficit Hyperactivity Disorder via Anti-Inflammatory Effects in Spontaneous Hypertensive Rats

**DOI:** 10.3389/fphar.2020.617581

**Published:** 2021-01-18

**Authors:** Yuchen Song, Haixia Yuan, Tianyi Chen, Manqi Lu, Shuang Lei, Xinmin Han

**Affiliations:** ^1^ Affiliated Hospital of Nanjing University of Chinese Medicine, Nanjing, China; ^2^ First Clinical Medical College, Nanjing University of Chinese Medicine, Nanjing, China; ^3^ Jiangsu Key Laboratory of Pediatric Respiratory Disease, Nanjing, China; ^4^ College of Traditional Chinese Medicine, Gansu University of Chinese Medicine, Gansu, China

**Keywords:** attention deficit hyperactivity disorder, An Shen Ding Zhi Ling, spontaneously hypertensive rat, neuroinflammation, blood-brain barrier, MAPK signaling pathway, NF-κB signaling pathway

## Abstract

Attention deficit hyperactivity disorder (ADHD) is a childhood-onset chronic neurobehavioral disorder, with multiple genetic and environmental risk factors. Chronic inflammation may be critical for the progression of ADHD. An Shen Ding Zhi Ling (ASDZL) decoction, a traditional Chinese medicine prescription, is clinically used in ADHD treatment. In this study, we investigated the effects and underlying anti-inflammatory mechanisms of ASDZL in young spontaneously hypertensive rats (SHRs), a widely used model of ADHD. SHRs were divided into the SHR model group (vehicle), atomoxetine group (4.56 mg/kg/day) and ASDZL group (21.25 g/kg/day), and orally administered for four weeks. Wistar Kyoto rats were used as controls (vehicle). We found that ASDZL significantly controlled hyperactivity and impulsivity, and improved spatial memory of SHRs in the open field test and Morris water maze test. ASDZL reduced the pro-inflammatory factors interleukin (IL)-1β, IL-4, IL-6, tumor necrosis factor (TNF)-α and monocyte chemoattractant protein (MCP)-1 and increased anti-inflammatory factor IL-10 in SHRs, and decreased the activation of microglia, astrocytes and mast cells in the prefrontal cortex (PFC) and hippocampus. Furthermore, the results indicated that ASDZL inhibited the neuroinflammatory response by protecting the integrity of the blood-brain barrier and suppressing the mitogen-activated protein kinase (MAPK) and nuclear factor (NF)-κB signaling pathways of SHRs. In conclusion, these findings revealed that ASDZL attenuated ADHD symptoms in SHRs by reducing neuroinflammation.

## Introduction

Attention deficit hyperactivity disorder (ADHD) is a chronic neurobehavioral disorder with a worldwide prevalence of 1.4–3.0%. The core symptoms of ADHD are inattention, motor hyperactivity and impulsivity, which affect the full lifecycle (Thapar and Cooper, 2016). Similar to other psychiatric disorders, interest in ADHD has increased over the past several decades in response to research into its pathogenesis and treatment. Genetic and environmental factors are considered risk factors for ADHD (Thapar et al., 2013). Studies in animal models have found that ADHD may be related to dopaminergic, noradrenergic and serotonergic neurotransmission (Russell, 2011). However, the exact biological mechanisms of ADHD are not yet fully elucidated, and there remain no neurobiological diagnostic markers.

Researches have proposed an association between ADHD and inflammatory mechanisms. Several studies have shown that patients displaying ADHD symptoms have abnormal serum cytokine levels compared with normal controls (O'Shea et al., 2014; Donfrancesco et al., 2016; Anand et al., 2017; Darwish et al., 2019). In addition, ADHD may have comorbidities with inflammatory and allergic disorders such as atopic eczema, asthma and atopic dermatitis (Feng et al., 2017; Miyazaki et al., 2017). Chronic pro-inflammatory immune dysregulation especially cellular mechanisms was considered to contribute to ADHD development (Verlaet et al., 2014). Imbalance between oxidants and antioxidants, catecholaminergic dysregulation, medications, genetic and environmental factors could be producing neuroinflammation which further increased the ADHD symptoms as a result (Corona, 2020). Besides, developmental or prenatal exposure to inflammation resulted in ADHD. And it presented accumulating evidence for a possible role of peripheral inflammation and neuroinflammation in the progression of ADHD (Dunn et al., 2019). However, there was no strong evidence to clarify the inflammatory mechanisms of ADHD. The neuroinflammatory mechanisms of other psychiatric diseases such as depression, Alzheimer's disease and autism spectrum disorder have already been extensively studied. Increased levels of pro-inflammatory factors, activation of innate immune cells in the brain such as microglia and mast cells, and blood-brain barrier (BBB) destruction have been found in these psychiatric disorders (Miller and Raison, 2016; Hendriksen et al., 2017; Pape et al., 2019). Mitogen-activated protein kinase (MAPK) and nuclear factor (NF)-κB signaling pathways play vital roles in neuroinflammation and the interaction between microglia, astrocytes and mast cells (Skaper et al., 2018). Besides, peripheral inflammation could generate several pro-inflammatory and neurotoxic mediators, causing neuroinflammation through the MAPK and NF-κB pathways (Kempuraj et al., 2017). We also proposed that ADHD may be a neuroinflammatory disease, related to the activation of immune cells in the brain based on literature review (Song et al., 2020b).

Efficacious medications such as methylphenidate and atomoxetine (ATX) are widely used, often in combination with complementary psychosocial approaches. However, their effectiveness has been questioned because they do not meet the broader and, especially, long-term clinical needs of many ADHD patients. Therefore, scientific and clinical studies are challenging current conceptions of the etiology and pathogenesis of ADHD that might alter future clinical approaches (Posner et al., 2020).

An Shen Ding Zhi Ling (ASDZL) is an empirical formula prescribed by Professor Xinmin Han for treating ADHD, which has been used for more than 20 years in Jiangsu Provincial Hospital of Traditional Chinese Medicine and included in Chinese Medical Clinical Pathway for Treating ADHD (Guo et al., 2015). ASDZL consists of Scutellariae Radix, Bupleuri Radix, Forsythiae Fructus, Curcumae Radix, Cassiae Semen, Bambusae Concretio Silicea, Uncariae Ramulus Cum Uncis, Rehmanniae Radix, Acori Tatarinowii Rhizoma, Angelicae Sinensis Radix, Alpiniae Oxyphyllae Fructus and Polygalae Radix. A clinical trial proved the efficacy and safety of ASDZL on children with ADHD (Han and Zhu, 2004). ASDZL has been shown to control ADHD symptoms by increasing dopamine (DA) levels in the brain and affecting the synthesis, transport and release of synaptosomes of SHRs (Zhou et al., 2017a; Song et al., 2018; Zhou et al., 2018). Besides, some herbal components of ASDZL, such as baicalin, saikosaponin A and catalpol were shown to have positive effects in the treatment of ADHD in our previous studies (Sun et al., 2017a; Yuan et al., 2019; Zhou et al., 2019).

However, the neuroinflammatory mechanisms of ADHD and the effect of ASDZL on the condition remain unclear. Prefrontal cortex (PFC) and hippocampus dysregulation has been found in ADHD patients and animal models (Spencer et al., 2015). Spontaneously hypertensive rat (SHR) is similar to ADHD in terms of etiology, biochemistry, symptomatology, and treatment, so it is a commonly used and well-studied model of ADHD. SHR fulfills the validation criteria and compares well with clinical cases of ADHD. SHR exhibits fundamental behavioral characteristics of ADHD (face validity). Research on candidate genes, neurotransmitter dysfunction, neuropathology and pharmacology strongly supports construct validity of SHR. In addition, SHR model has demonstrable predictive validity for predicting aspects of ADHD behavior, genetics, and neurobiology previously unknown. And SHR is a genetic model and bred from progenitor Wistar Kyoto (WKY) rat (Sagvolden et al., 2005; Aparicio et al., 2019). Therefore, the present study sought to use SHR as ADHD model and WKY rat as normal control to investigate the possible underlying neuroinflammatory mechanisms of ADHD and explore the effects of ASDZL on behavioral performance, as well as inflammatory factors, immune cells in the brain, BBB and inflammation-related MAPK and NF-κB signaling pathways in the PFC and hippocampus of SHRs.

## Materials and Methods

### Preparation of ASDZL Decoction

The ASDZL decoction was composed of 12 herbs, as shown in Table 1. All the herbs were purchased from the Affiliated Hospital of Nanjing University of Chinese Medicine and authenticated by Professor Jian-Guo Chao of Nanjing University of Chinese Medicine.

**TABLE 1:** Composition of ASDZL.

Component	Chinese name	Family	Part used	Origin (PR China)/Batch number	Voucher number	Weight (*g*)
*Scutellaria baicalensis* Georgi	Huang Qin	Lamiaceae	Roots	He Bei/190501	NZY-Han-2019001	10
*Bupleurum chinense* DC.	Cu Chai Hu	Apiaceae	Roots	Shan Xi/20190801-01	NZY-Han-2019002	6
*Forsythia suspensa* (Thunb.) Vahl	Lian Qiao	Oleaceae	Fruits	Shan Xi/190501	NZY-Han-2019003	10
Curcuma aromatica Salisb	Yu Jin	Zingiberaceae	Roots	Zhe Jiang/190702	NZY-Han-2019004	10
Senna obtusifolia (L.) H.S.Irwin & Barneby	Jue Ming Zi	Leguminosae	Seeds	An Hui/190701	NZY-Han-2019005	10
*Bambusa textilis* McClure	Tian Zhu Huang	Poaceae	Dried lumps of stem endocrine fluid	Guang Dong/190527	NZY-Han-2019006	10
Uncaria rhynchophylla (Miq.) Miq.	Gou Teng	Rubiaceae	stems and branches with hooks	Hu Nan/20190603-1	NZY-Han-2019007	10
Rehmannia glutinosa (Gaertn.) DC.	Sheng Di Huang	Plantaginaceae	Roots	He Nan/19071917	NZY-Han-2019008	10
Acorus calamus var. angustatus Besser	Shi Chang Pu	Acoraceae	Rhizome	An Hui/190401	NZY-Han-2019009	10
*Angelica sinensis* (Oliv.) Diels	Dang Gui	Apiaceae	Roots	Gan Su/19090214	NZY-Han-2019010	10
*Alpinia oxyphylla* Miq	Yi Zhi Ren	Zingiberaceae	Fruits	Hai Nan/190701	NZY-Han-2019011	10
*Polygala tenuifolia* Willd	Yuan Zhi	Polygalaceae	Roots	Shan Xi/190901	NZY-Han-2019012	6

The herbs in ASDZL were mixed and soaked in 8-time volume of distilled water (v/w) for 1 h. Then the herbs were boiled twice in 8-time volume of distilled water (v/w) for 1 h each time. The supernatants acquired from both decoctions were pooled and concentrated to 0.71 g/ml of crude herbs using rotavapor (Buchi, Switzerland).

### Qualitative Analysis of Components of ASDZL Water Extract

**FIGURE 1:**
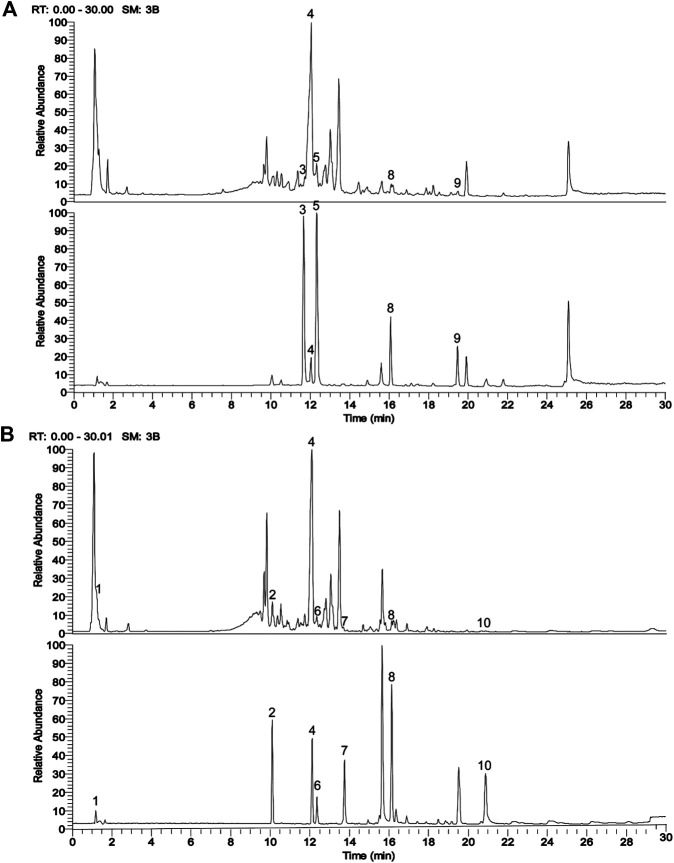
UPLC chromatograms analysis of ASDZL with the representative active ingredients. **(A)** The total ion chromatogram (TIC) in positive ion mode (above, ASDZL; below, standards); **(B)** The TIC in negative ion mode (above, ASDZL; below, standards).

A Waters Aquity H-Clas ultra-high performance liquid chromatograph was used and chromatographic separation was achieved by the AQUITY UPLC HSS T3 chromatographic column (2.1 × 100 mm, 1.8 μm). The column oven temperature was set at 25°C and the mobile phases consisted of 0.1% formic acid water (A) and 0.1% formic acid acetonitrile (B). The elution conditions were as follows: 0 min, 10% B; 3 min, 10% B; 26 min, 90% B; 30 min, 10% B, at the flow rate of 0.2 ml/min. The linear trap quadrupole (LTQ) ORBITRAP XL tandem mass spectrometer (MS) was applied and MS conditions were: ion source, heated electrospray ionization (HESI); scan ranges, 100–1,000 m/z; collision energy, 35. The positive ionization mode used the following parameters: heater temperature, 300°C; sheath gas flow rate, 45 arb; aux gas flow rate, 10 arb; spray voltage, 4 kV; capillary temperature, 300°C, capillary voltage, 35 V; tube lens, 110 V. The negative ionization mode used the following parameters: heater temperature, 300°C; sheath gas flow rate, 45 arb; aux gas flow rate, 10 arb; spray voltage, 4 kV; capillary temperature, 300°C; capillary voltage, −35 V; tube lens, −110.00 V. The reference standards used were Baicalin, Saikosaponin A, Rhynchophylline, Isorhynchophylline, Emodin (Chengdu Pufei De Biotech Co. Ltd., China), Curcumin, Quercetin, Phyllyrin, Catalpol, Acteoside (Chengdu Herbpurity Co. Ltd., China), and the detailed chemical information of ASDZL was shown in Figure 1 and Table 2.

**TABLE 2:** The chemical components identified from ASDZL.

No	Components	Formula	Adduct	MW (Da)	Standard RT (min)	ASDZL RT (min)	ASDZL m/z	ppm
1	Catalpol	C_15_H_22_O_10_	[M + FA-H]^-^	407.11840	1.18	1.15	407.11945	2.573
2	Acteoside	C_26_H_36_O_15_	[M-H]^-^	623.19704	10.11	10.1	623.19537	−1.677
3	Isorhynchophylline	C_22_H_28_N_2_O_4_	[M + H]^+^	385.21218	11.66	11.69	385.21219	−0.451
4	Baicalin	C_21_H_18_O_11_	[M + H]^+^	447.09219	12.03	12.03	447.09259	0.9
			[M-H]^-^	445.07654	12.12	12.11	445.07748	2.117
5	Rhynchophylline	C_22_H_28_N_2_O_4_	[M + H]^+^	385.21218	12.33	12.36	385.21201	0.016
6	Phyllyrin	C_27_H_34_O_11_	[M + FA-H]^-^	579.20722	12.35	12.36	579.20593	−2.223
7	Quercetin	C_15_H_10_O_7_	[M-H]^-^	301.03427	13.74	13.73	302.03552	4.122
8	Saikosaponin A	C_42_H_68_O_13_	[M + Na]^+^	803.45521	16.06	16.09	803.45496	−0.016
			[M + FA-H]^-^	825.46310	16.13	16.14	825.43610	−0.106
9	Curcumin	C_21_H_20_O_6_	[M + H]^+^	369.13326	19.44	19.45	369.13293	−0.907
10	Emodin	C_15_H_10_O_5_	[M-H]^-^	269.04444	20.86	20.87	269.04495	1.859

### Animal Experiments

Three-week-old male WKY rats (*n* = 10) and SHRs (*n* = 30) were purchased from Beijing Vital River Laboratory Animal Technology Co., Ltd. (Beijing, China, SCXK (Jing) 2016-0006) and maintained on a 12-h light/dark cycle under the temperature of 23 ± 2°C and relative humidity of 50–60% in the experimental animal center of Nanjing University of Chinese Medicine. All rats received adaptive feeding for 1 week prior to administration. All procedures were carried out in accordance with the National Institute of Health Guidelines for Laboratory Animals, and were approved by the Animal Ethics Committee of Nanjing University of Chinese Medicine (No. 201910A041). The weight changes of rats in each group were shown in Table 3.

**TABLE 3:** Weekly weight changes of rats in each group.

Group	Day 0 (g)	Day 7 (g)	Day 14 (g)	Day 21 (g)	Day 28 (g)
WKY	56.4 ± 4.6	91.8 ± 6.8	130.6 ± 3.7	168.4 ± 2.9	200.2 ± 6.1
SHR	57.7 ± 3.4	93.5 ± 3.3	129.9 ± 1.9	167.4 ± 3.8	202.5 ± 6.5
ATX	59.7 ± 2.8	94.1 ± 5.6	129.7 ± 6.4	168.5 ± 7.0	204.5 ± 9.6
ASZDZL	59.9 ± 4.0	95.1 ± 5.7	131.7 ± 9.0	168.0 ± 12.2	200.6 ± 7.9

### Drug Administration

**FIGURE 2:**
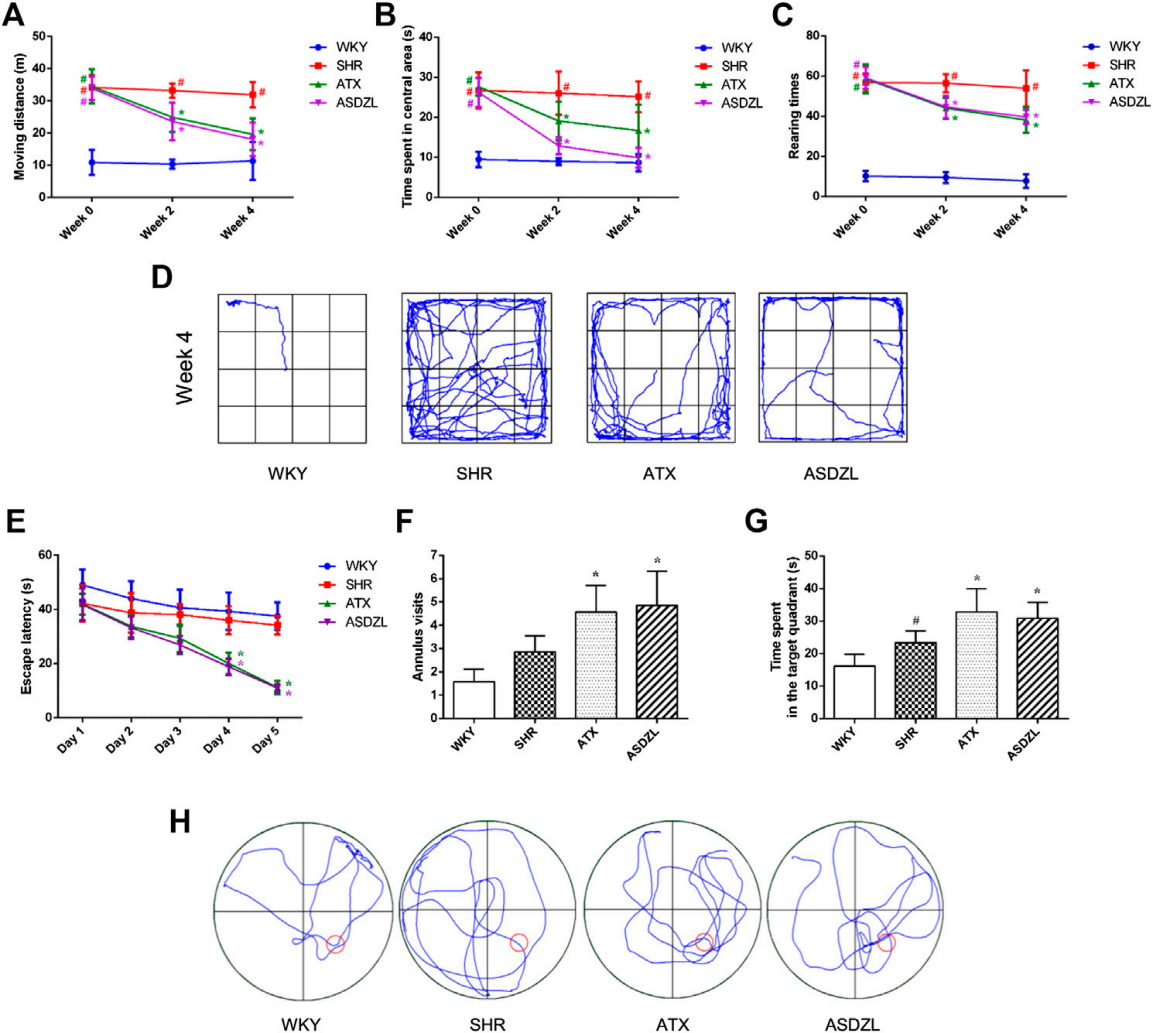
Behavioral performance in OFT and MWM. **(A)** Moving distance in OFT (m); **(B)** Time spent in the central area in OFT (s); **(C)** Rearing number in OFT; **(D)** Representative trajectories of each group in OFT; **(E)** Escape latencies in spatial acquisition trial of MWM; **(F)** Number of annulus visits in probe trial of MWM; **(G)** Time spent in the target quadrant (s) in probe trial of MWM; **(H)** Representative trajectories of each group in MWM. All data are presented as mean ± SD, *n* = 10 for each group. ^*#*^ *p* < 0.05 SHR vs. WKY group, *p < 0.05, compared to SHR group.

ATX, a norepinephrine (NE) reuptake inhibitor and commonly used drug for ADHD treatment (Thapar and Cooper, 2016) was used as a positive control drug for ASDZL. ATX obtained from Lilly Del Caribe Inc., US (C679184A) was dissolved in distilled water to a concentration of 0.152 mg/ml. After adaptive feeding, the SHRs were randomly divided into the three groups (*n* = 10 per group): SHR group (model group), ATX group (4.56 mg/kg), ASDZL (21.25 g/kg) group, and WKY rats were used as the control group. There were no significant differences in the weight gain of rats among the groups (Table 3), and no significant differences in moving distance in the open field test (OFT) among model group and medication groups before the experiments (Figure 2A). The WKY (control) and SHR (model) groups were oral gavaged with distilled water while the ATX and ASDZL groups were gavaged with the corresponding drugs twice a day (08:00–09:00 am 14:00–15:00 pm) for 4 weeks. The dosage for the rats was calculated according to the human and rat body surface area. The rats were daily weighed before gavage, and the administration volume was determined according to the body weight (1.5 ml/100 g). All drugs were brought to 23 ± 2°C before administration. After behavioral tests, the rats were anesthetized and sacrificed. Blood samples were obtained from abdominal aorta and centrifuged at 850 g for 10 min at 4°C. The brain tissues were immediately collected and isolated for experiments or snap-frozen in liquid nitrogen then stored at −80°C for further study.

### Behavioral Tests

The OFT was conducted at 0, 2 and 4 weeks of treatment, and the Morris Water Maze (MWM) test was performed after 3 weeks of treatment. Behavioral tests were performed based on previously established methods (Yuan et al., 2019) with minor modifications.

### Open Field Test (OFT)

The OFT is used to assess behavioral performance in ADHD models (Archer, 1973). The open field arena used in this study consisted of a black iron box (100 × 100 × 40 cm) and the total area was divided into 16 equal squares of 25 × 25 cm (with a central area of 50 × 50 cm and peripheral area of 25 cm on each side). The movement of each rat was observed for 5 min. Moving distance (*m*), time spent in the central area (s) and rearing times were recorded using Easy-Tracking System (SLY-ETS Version 1.66, Beijing Sunny Instruments Co. Ltd.). The OFT was conducted in a quiet environment from 8:00–12:00 am. The scent marks were removed between tests.

### Morris Water Maze (MWM) Test

The MWM test is widely used for assessing the learning and memory abilities. The MWM apparatus consisted of a circular black iron tank (150 cm in diameter and 50 cm in depth) filled with water (24 ± 2°C), a video camera above the arena and a computerized tracking system. A circular black platform with a rough surface (12 cm in diameter) was submerged approximately 1 cm below the water surface. Spatial acquisition trials were conducted during day 1–5 with four trials per day, and the trials were conducted from four positions. Each rat had 60 s to find the platform. Rats that failed to locate the platform were guided there and allowed to rest on it for 10 s. The probe trials were conducted on day 6 without the platform. The escape latency (s) in spatial acquisition trials, annulus visits and time spent in the target (southeast) quadrant (s) in the probe trials were recorded using the Easy-Tracking System.

### Enzyme-Linked Immunosorbent Assay (ELISA)

The concentrations of interleukin (IL)-1β, IL-4, IL-6, IL-10, tumor necrosis factor (TNF)-α and monocyte chemoattractant protein (MCP)-1 in the serum, PFC and hippocampus were measured using ELISA kits purchased from MULTI SCIENCES, China or Shanghai WESTANG, China, according to the manufacturer's protocol. In addition, about 6–8 ml blood can be collected from abdominal aorta of each rat and 40 or 100 μL serum was needed for each target protein tested by ELISA.

### Electron Microscopy

The PFC (1 × 1 × 1 mm^3^) of each rat was submerged into electron microscope fixing solution at 4°C for 3 h, washed three times with PBS (pH 7.4), fixed with 4% osmium acid, and washed with PBS again. Then the samples were dehydrated in gradient alcohol washes, embedded in Epon 812 and polymerized at 60°C for 48 h. The samples were sliced into ultrathin sections of 60–80 nm, which were negatively stained with uranyl acetate and lead citrate and examined using a JEM-1200EX electron microscope to observe the ultrastructure of the BBB in different groups.

### Immunohistochemistry and Immunofluorescence

Immunohistochemistry and immunofluorescence were performed based on previously established methods (Yuan et al., 2019). The PFC and hippocampus in each group were fixed with 4% paraformaldehyde, then embedded in paraffin, cut into sections of 3–4 μm in thickness. The brain sections from each group were analyzed by immunohistochemistry to explore the expressions of tryptase, and by immunofluorescence to assess the expression of ionized calcium binding adaptor molecule 1 (Iba1) and glial fibrillary acid protein (GFAP). The nuclei were counterstained with DAPI in immunofluorescence staining. Images of the PFC and hippocampus were captured using a fluorescence microscope (Olympus BX43, Tokyo, Japan) at 200× (for tryptase) or 400× (for Iba1 and GFAP) magnification. The ImageJ 1.52v software was used to analyze number of positive stained cells and average optical density (AOD) and in the sections.

### Toluidine Blue

The PFC and hippocampus tissues of each rat were fixed with 4% paraformaldehyde for 48 h at 4°C, paraffin-embedded, dewaxing to water, and sliced to 4 μm and stained with toluidine blue. Photographing was conducted under the microscope at a 200 times the field of view using Nikon ECLIPSE E100 microscope (Nikon, Tokyo, Japan). The ImageJ 1.52v software was used to analyze number of positive stained cells in the sections.

### Western Blotting

The proteins were extracted from the PFC or hippocampus tissue in each group and measured by the BCA protein assay kit (Thermo Scientific, USA). Equal amounts of proteins were separated with SDS-PAGE, and transferred to polyvinylidene difluoride membranes (0.45 μm, Millipore, USA). The membranes were blotted overnight at 4°C with primary antibodies: anti-ZO-1 (1:1,000), occludin (1:1,000), p-p38 (1:1,000), p38 (1:1,000), p-c-Jun N-terminal kinase (JNK) (1:1,000), JNK (1:1,000), p-inhibitor of NF-κB (IκBα) (1:1,000), IκBα (1:1,000), p-NF-κB (1:1,000), NF-κB (1:1,000), and GAPDH (1:10,000). Following incubation with secondary antibodies. The ChemiDoc™ MP Imaging system (Bio-Rad Co., United States) was used to quantify the protein bands with the Image Lab software (Bio-Rad Co., United States). Each experiment was repeated at least three times.

### Statistical Analysis

Data were presented as mean ± standard deviation (SD) and were analyzed using one-way analysis of variance (ANOVA), followed by Dunnett’s *post hoc* test to determine the statistical significance, which was performed by GraphPad Prism 6.0 (GraphPad Software Inc., San Diego, CA, United States). Differences with *p* < 0.05 were considered statistically significant.

## Results

### Qualitative Analysis of the Major Chemical Constituents in Water Extract of ASDZL Decoction

The main components of ASDZL were determined by UPLC and compared with the reference standard solution. A total of 10 chemical components in ASDZL were determined, namely Catalpol, Acteoside, Isorhynchophylline, Baicalin, Rhynchophylline, Phyllyrin, Quercetin, Saikosaponin A, Curcumin and Emodin (Figure 1 and Table 2).

### Effect of ASDZL on Behavior of SHRs in the OFT

ASDZL had no negative effect on the weight changes and development of rats, as shown in Table 3. The data showed no significant differences in moving distance, time spent in central area, and rearing times before administration among SHR, ATX, and ASDZL groups. Moreover, these three groups showed longer moving distance, longer time spent in central area and more rearing times compared with WKY rats (Figures 2A–C). After 4 weeks treatment, the ATX and ASDZL groups showed a significant decrease in moving distance, time spent in central area and rearing times compared with the SHR (Figures 2A–C). The representative pathways of all groups after 4 weeks of drug administration are shown in Figure 2D.

### Effect of ASDZL on Behavior of SHRs in the MWM Test

The data revealed that there was a decrease in escape latencies of all four groups during the spatial acquisition trials. The ATX and ASDZL groups spent less time to locate the escape platform compared with SHR group on the day 4 and 5 (Figure 2E). The SHR group made more visits to the annulus than the WKY group without the platform, but there was no significant difference (Figure 2F). The number of annulus visits of both ATX and ASDZL groups was significantly greater than that of SHR group (Figure 2F). In addition, the SHR group spent more time in the target quadrant than the WKY group, while the time spent in ATX and ASDZL groups was longer than that in SHR group (Figure 2G). The representative swimming patterns of all groups during probe trials are shown in Figure 2H.

### Effect of ASDZL on Inflammatory Factors in Serum, PFC and Hippocampus

**FIGURE 3:**
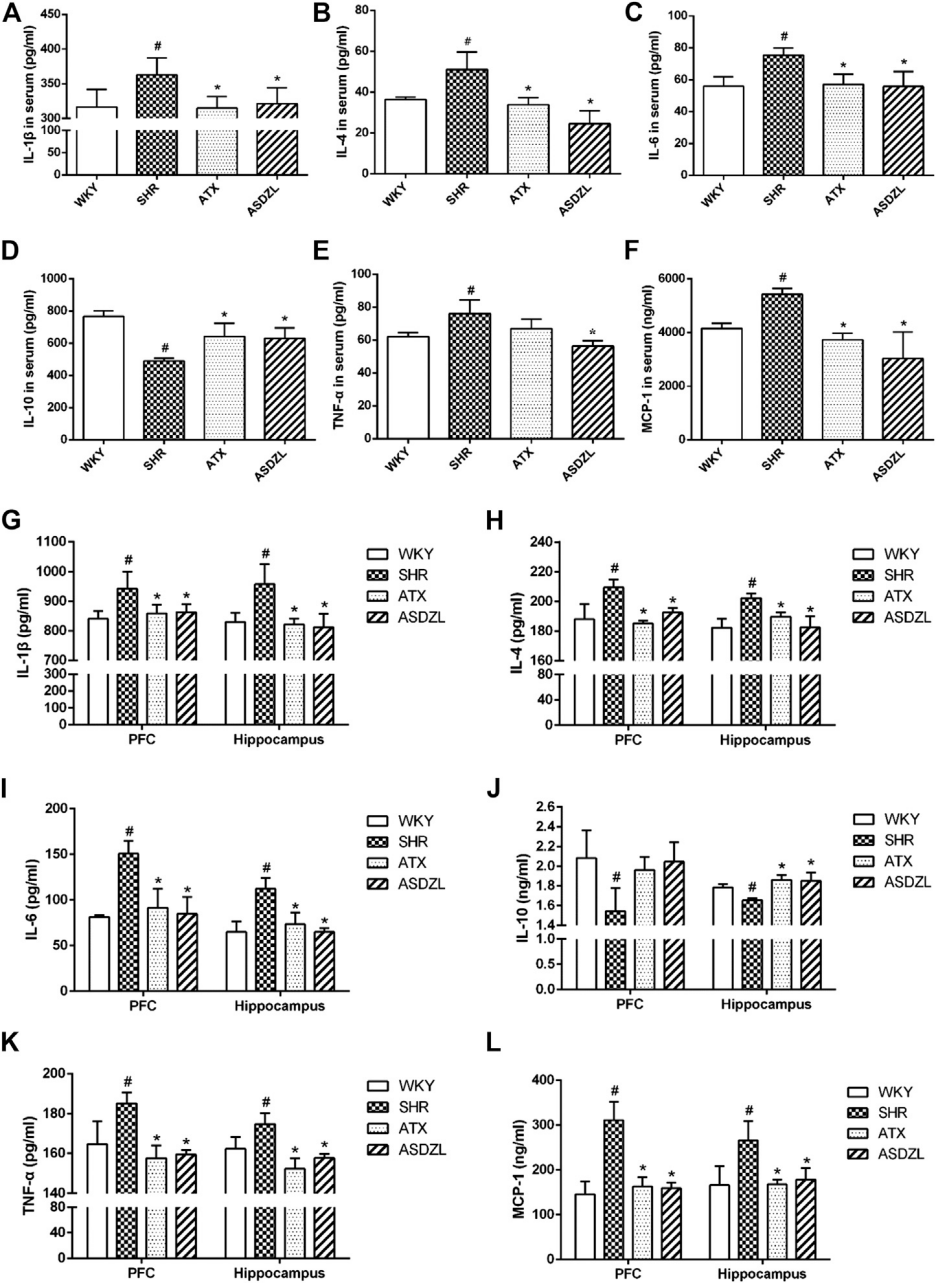
Effect of ASDZL on IL-1β, IL-4, IL-6, IL-10, TNF-α and MCP-1. (A–F) Levels of IL-1β, IL-4, IL-6, IL-10, TNF-α and MCP-1 in the serum. (G–L) Levels of IL-1β, IL-4, IL-6, IL-10, TNF-α and MCP-1 in the PFC and hippocampus tissues. Data were presented as the mean ± SD, *n* = four to six for each group. ^*#*^ *p* < 0.05 SHR vs. WKY group, *p < 0.05, compared to SHR group.

IL-1β, IL-4, IL-6, TNF-α and MCP-1 are pro-inflammatory factors commonly used to evaluate inflammatory responses, and IL-10 is an anti-inflammatory factor (Potvin et al., 2008). As shown in Figure 3, the levels of IL-1β, IL-4, IL-6, TNF-α and MCP-1 in the serum of SHR rats were significantly higher than those in the serum of WKY rats, and these factors were effectively decreased (Figures 3A–C,F) or showed a tendency to decrease (*p* > 0.05, Figures 3E) after ATX or ASDZL administration. Moreover, the levels of IL-1β, IL-4, IL-6, TNF-α and MCP-1 in the PFC and hippocampus of SHRs were also increased, and ATX and ASDZL reversed the levels of these pro-inflammatory factors (Figures 3G–I,K,L). The levels of IL-10 in the serum, PFC and hippocampus of SHRs were significantly lower than those in the WKY rats, and IL-10 levels in the serum and hippocampus of ATX and ASDZL groups were effectively increased compared with SHR group (Figures 3D,J). Furthermore, a tendency to increase was shown in the levels of IL-10 in the PFC of ATX and ASDZL groups compared with the SHR group (Figure 3J).

### Effect of ASDZL on Microglia, Astrocytes and Mast Cells

**FIGURE 4:**
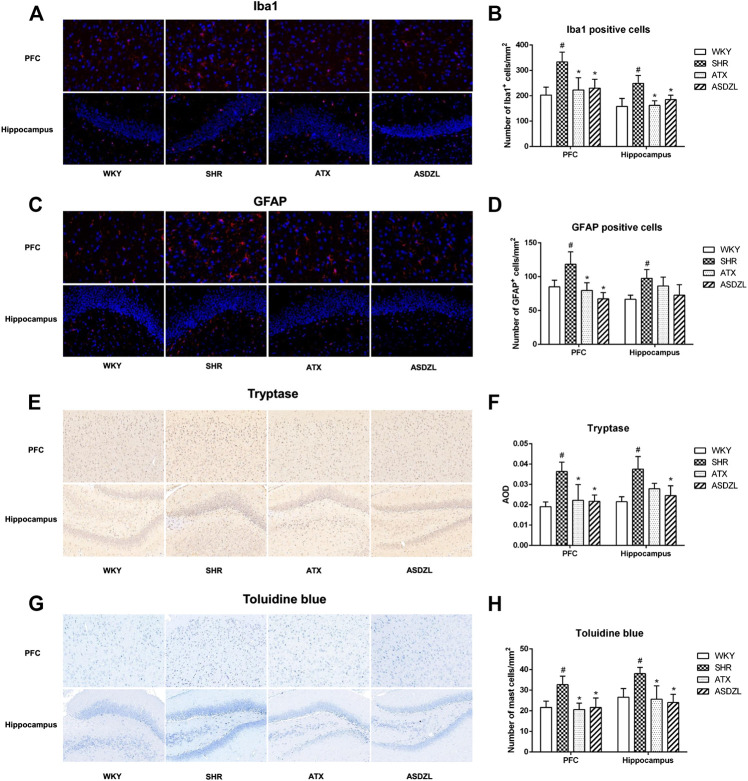
Effect of ASDZL on microglia, astrocytes and mast cells. **(A)** Representative images of Iba1-immunopositive microglia (red) with DAPI (blue) staining from PFC and hippocampus; **(B)** Quantification of Iba1+ cells in the PFC and hippocampus; **(C)** Representative images of GFAP-immunopositive astrocytes (red) with DAPI (blue) staining from the PFC and hippocampus; **(D)** Quantification of GFAP + cells in the PFC and hippocampus; **(E)** Immunostaining for tryptase in PFC and hippocampus tissues; **(F)** Relative protein expressions of tryptase were quantified by AOD; **(G)** Mast cells in the PFC and hippocampus were stained with toluidine blue (TB); **(H)** Quantification of activated mast cells in the PFC and hippocampus. Data were presented as the mean ± SD, *n* = 3 for each group. ^*#*^ *p* < 0.05 SHR vs. WKY group, *p < 0.05, compared to SHR group.

As shown in Figure 4, microglia, astrocytes and mast cells were activated and infiltrated in the SHR group. A higher number of Iba1+ microglial cells (Figures 4A,B), GFAP + astrocytes (Figures 4C,D) and tryptase + mast cells (Figures 4E,F) were observed in the PFC and hippocampus tissues of the rats in the SHR group compared with the WKY group. Toluidine blue staining showed comparable results to those of immunohistochemistry for tryptase (Figures 4G,H). And we confirmed that after ATX or ASDZL treatment, the infiltration of microglia, astrocytes and mast cells in PFC and hippocampus of SHRs was inhibited.

### Effect of ASDZL on the BBB of SHRs

**FIGURE 5:**
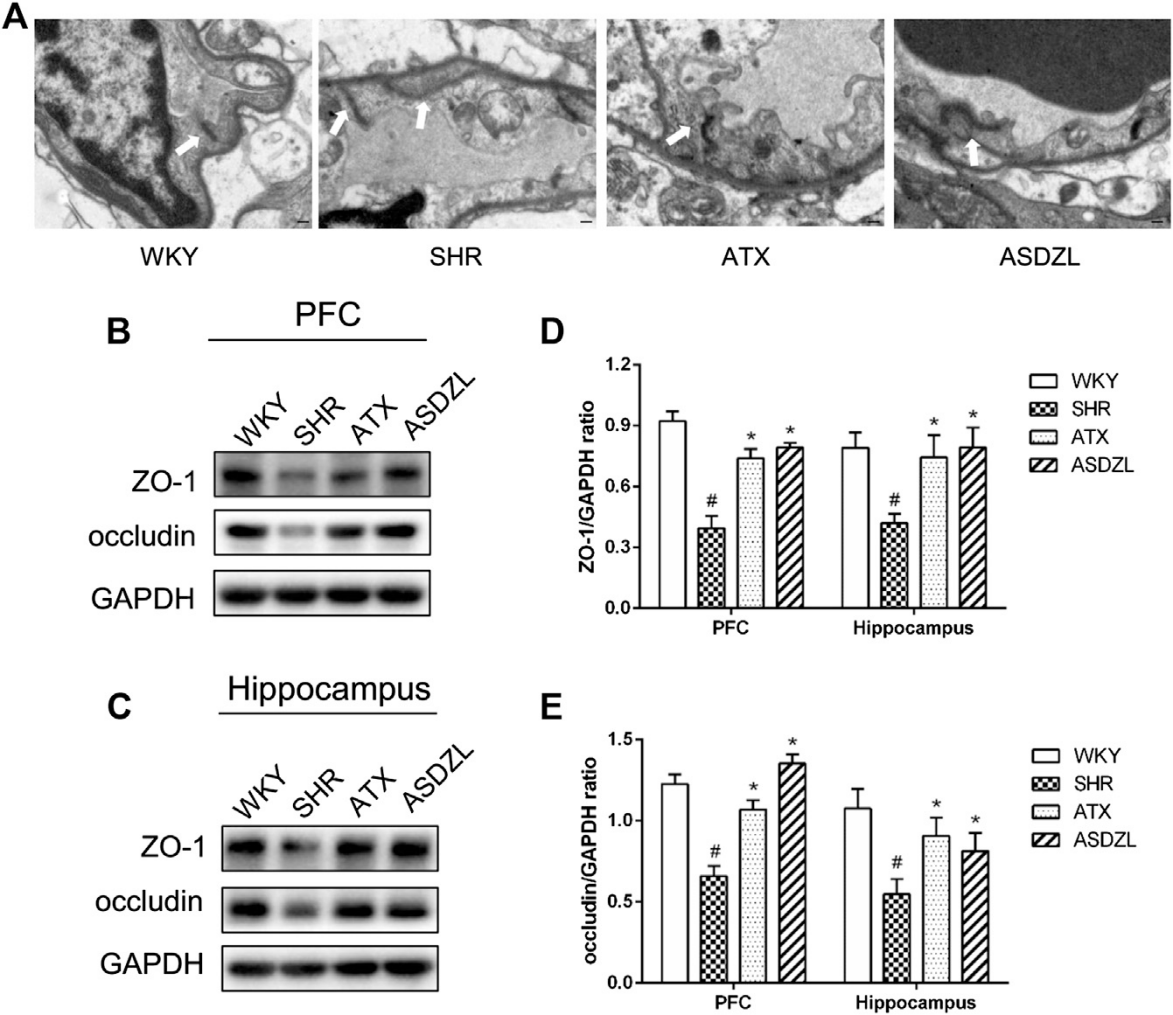
Effect of ASDZL on the BBB. **(A)** Typical images of the TJs examined by electron microscopy (EM), and the arrows point to the TJs; **(B)** Western blot analysis of ZO-1 and occludin levels in PFC; **(C)** Western blot analysis of ZO-1 and occludin levels in hippocampus; **(D)**, **(E)** Relative levels of ZO-1 and occludin in PFC and hippocampus (fold change relative to GAPDH level). Data are expressed as the mean ± SD, *n* = 3 for each group. *p < 0.05, SHR vs. WKY group; ^*#*^ *p* < 0.05, compared to SHR group. Scale bar is 1.0 μm.

As shown in Figure 5A, tight junctions (TJs) between adjacent endothelial cells (ECs) in the PFC of WKY group were integral and normal, with typical thickened dark lines along the cell membrane. The gap between adjacent ECs in the PFC of SHR group slightly increased compared with WKY group, while ATX and ASDZL administration restored the integrity of TJs (Figure 5A). In addition, the expressions of occludin and ZO-1 were significantly lower in the PFC and hippocampus of SHR group, and occludin and ZO-1 were higher in ATX and ASDZL groups (Figures 5B–E). The results indicated that ASDZL alleviated the BBB breakdown in the PFC and hippocampus of SHRs.

### Effect of ASDZL on MAPK and NF-κB Pathways

**FIGURE 6:**
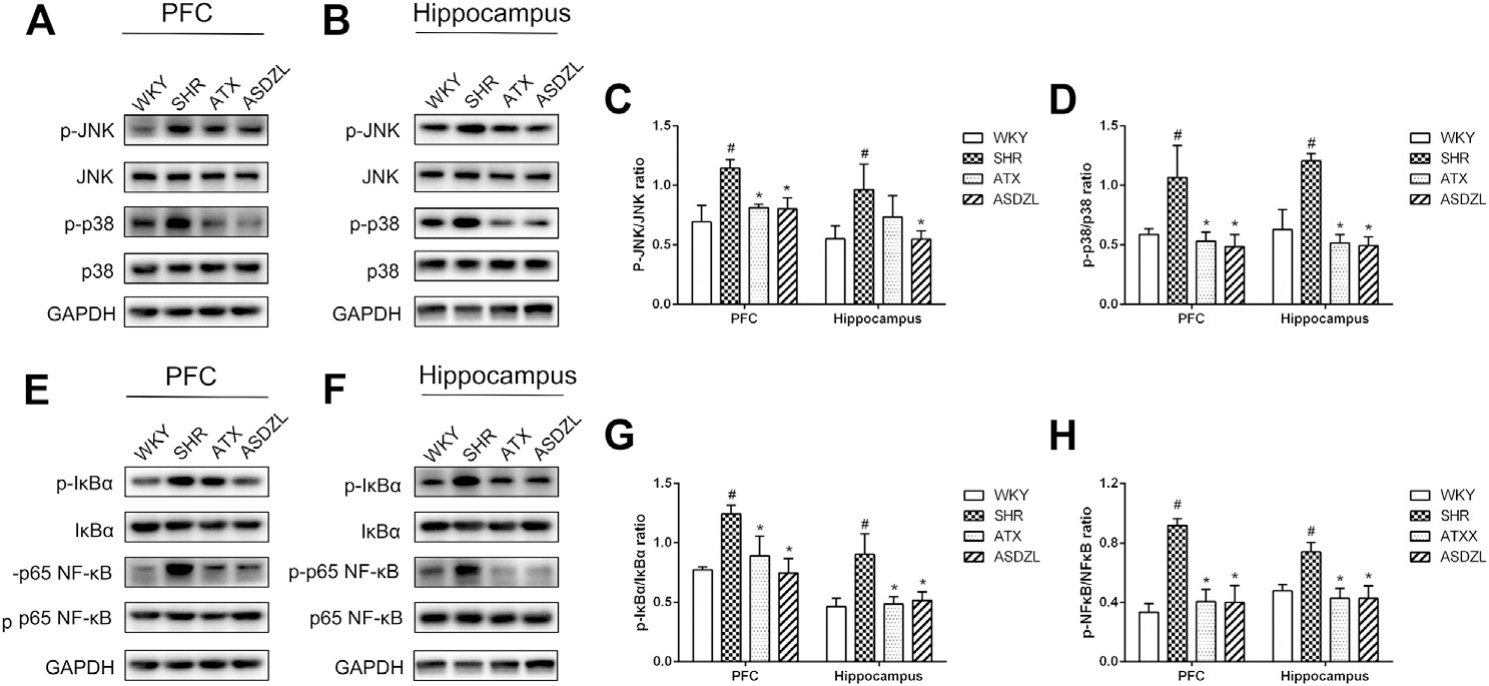
Effect of ASDZL on MAPK and NF-κB signaling pathways. **(A)** Western blot analysis of *p*-JNK, JNK, p-p38 and p38 levels in PFC; **(B)** Western blot analysis of *p*-JNK, JNK, p-p38 and p38 levels in hippocampus; **(C)**, **(D)** Relative levels of *p*-JNK and p-p38 in PFC and hippocampus; **(E)** Western blot analysis of *p*-IκBα, IκBα, p-NF-κB and NF-κB levels in PFC; **(F)** Western blot analysis of *p*-IκBα, IκBα, p-NF-κB and NF-κB levels in hippocampus; **(G)–(H)** Relative levels of *p*-IκBα and p-NF-κB in PFC and hippocampus. Data are expressed as the mean ± SD, *n* = 3 for each group. *p < 0.05, SHR vs. WKY group; ^*#*^ *p* < 0.05, compared to SHR group.

To evaluate the effect of ASDZL on inflammation-related signaling pathways, we measured the phosphorylation and total levels of proteins in the MAPK and NF-κB pathways. The p-p38 MAPK, *p*-JNK, *p*-IκBα and p-p65 NF-κB levels were significantly increased in the PFC and hippocampus of SHRs, while their total levels did not obviously change. And the p-p38 MAPK, *p*-JNK, *p*-IκBα and p-p65 NF-κB levels in ATX and ASDZL groups were significantly decreased compared with those in SHR group after 4 weeks of gavage treatment (Figures 6A–H). These results demonstrated that ASDZL inhibited the phosphorylation of the MAPK and NF-κB signaling pathways.

## Discussion

ADHD is a chronic behavioral disorder with unclear pathogenesis, and it usually requires long-term drug use to control the hyperactive, impulsive and inattentive symptoms. However, ATX and MPH, which have been the most widely used drugs for ADHD treatment have some potential side effects, such as irritability, growth restriction, gastrointestinal symptoms, sleep disturbances and drowsiness (Thapar and Cooper, 2016). Therefore, more effective and safe drugs need to be developed. In the present study, we evaluated the role of the traditional Chinese medicine prescription ASDZL on the behavioral performance and neuroinflammatory mechanisms of ADHD. This study provided new insights and theoretical bases for the research and development of therapeutic drugs for ADHD.

The effect of ASDZL on behavior and neuroinflammation of the ADHD model, young SHR were studied by behavioral, histomorphological and molecular methods. We measured weight changes in rats to assess the effect of ASDZL on their growth and development. The results showed that ASDZL did not affect the weight changes of SHRs, indicating it was safe for gavage. The OFT has been applied to evaluate hyperactive behavior in ADHD while the MWM has been widely used to assess the learning and memory abilities of SHRs (Majdak et al., 2014). As in previous studies, the SHRs in this study showed increased moving distance, time spent in central area and rearing times, which represented typical hyperactivity and inattentive symptoms compared with WKY rats, as shown in Figure 2. Rats in the ATX and ASDZL groups showed significant reductions in moving distance, time spent in central area and rearing times. The typical trajectories showed that the rats in ATX and ASDZL groups presented significant thigmotaxis movement loci, while the SHR group displayed a chaotic movement trajectory. Results of the MWM test showed that rats in ATX and ASDZL groups exhibited better learning and memory ability, with shorter escape latency, more annulus visits and longer time spent in the target quadrant, compared with rats in SHR group. However, it can be noticed that the behavioral performance of rats in SHR group was better than that of WKY rats in the MWM test. This observation may be interpreted that the SHRs showed faster swimming speed than the WKY rats, hence SHRs spent less time locating the platform (Ferguson and Cada, 2004). Thus, our results revealed that ASDZL controlled the core symptoms of ADHD in SHRs.

Inflammation is one of the main causes of the central nervous system (CNS) disorders and has been thought to be related to ADHD. Previous studies found abnormal expression of inflammatory factors in ADHD patients (O'Shea et al., 2014; Rivera et al., 2015; Donfrancesco et al., 2016; Anand et al., 2017; Darwish et al., 2019; Donfrancesco et al., 2020). However, investigation on inflammatory biomarkers has not showed consistent conclusions mainly because of the small sample sizes used and high heterogeneity among biomarkers (Leffa et al., 2018). In addition, a study of the animal model indicated elevation of serum and tissue contents of cytokines, chemokines and oxidative stress markers in SHRs and the neurological and immune systems had a synergistic effect in ADHD pathogenesis (Kozlowska et al., 2019). Inflammatory cytokines or other inflammatory stimuli (e.g., IL-1β, IL-4, IL-6, TNF-α, and MCP-1) induce depressive symptoms or other psychiatric disorders, and these factors may also predict the development of these disorders (Miller and Raison, 2016; Miller, 2020). Although some cytokines such as IL-6 play a bidirectional role under physiological conditions, an increase in their levels may produce neurotoxicity during inflammation (Vallieres et al., 2002). In this study, we determined the pro-inflammatory and anti-inflammatory cytokine and chemokine levels in the PFC, hippocampus and serum of rats. Compared with the SHR group, treatment with ASDZL significantly inhibited IL-1β, IL-4, IL-6, TNF-α, and MCP-1 in the serum, PFC and hippocampus and promoted the secretion of IL-10. Moreover, microglia, the resident immune cells in the CNS, play an important role in protecting against CNS injuries and maintaining brain homeostasis (Hu et al., 2014). Activated microglia are usually marked by Iba1, and GFAP is used to mark astrocytes. According to our previous research, mast cell-mediated neuroinflammation play a role in ADHD while the interaction between glia and mast cells may aggravate inflammatory responses in ADHD (Song et al., 2020). Toluidine blue is often used to evaluate the expression of mast cells, and tryptase is secreted from activated mast cells (Irani and Schwartz, 1994). The activation of microglia, astrocytes and mast cells was observed in the PFC and hippocampus of SHRs, whereas ASDZL attenuated the changes in the immune cells in the present study. These findings suggested that ASDZL reduced inflammation in the animal model of ADHD.

Healthy cerebral blood vessels have a functional BBB which protects the surrounding brain parenchyma from the invasion of immune cells and deleterious stimuli in the blood (Skaper et al., 2017). The ECs in the brain are the site of the BBB, which are characterized by TJs that are formed mainly by the claudin family members and occludin, which are linked to the cytoskeleton with the ZO family members (e.g., ZO-1), contributing to its integrity (Engelhardt and Liebner, 2014). In this study, changes in inflammatory factors of both peripheral and CNS were observed, suggesting BBB injury in SHRs. Interneurons and perivascular microglia contact ECs, pericytes and astrocytes to form a neuro-vascular unit, together with surrounding mast cells and render important effects on the integrity and function of the BBB (Liebner et al., 2018). The complex interplay between glia and mast cells induces and aggravates the inflammatory process and affects the BBB (Skaper, 2016). Some inflammatory cytokines such as TNF-α, IL-1β and IL-6 downregulate TJs, causing increased BBB permeability (Rochfort and Cummins, 2015). Once neuroinflammation occurs, immune cells in the brain are activated and inflammatory factors are released, resulting in the destruction of the BBB, further promoting the activity of neurotoxic substances and immune cells, which in turn aggravates neuroinflammation and lead to an increase release of inflammatory factors. The breakdown of BBB has been implicated in the initiation and progression of diseases such as multiple sclerosis, stroke and Alzheimer's disease (Daneman and Prat, 2015; Ribatti, 2015). It was observed in our study that TJs of BBB were destroyed in the SHR group, while ATX and ASDZL protected the integrity of the BBB.

MAPKs are important for intracellular signal transduction and play vital roles in regulating neuroplasticity and inflammatory responses, and p38 MAPK and JNK are important members of the MAPK family, which have been considered as anti-inflammation targets (Ji et al., 2009; Feng et al., 2016). Inhibition of the MAPK signaling pathway has been shown to reduce neuroinflammation in animal models of different diseases (Crown et al., 2008; Lee et al., 2011). NF-κB is an important nuclear transcription factor and NF-κB pathway, which is the downstream pathway of MAPK is a classic pathway related to inflammation and activation of immune cells in the brain. Its activation promotes the production of pro-inflammatory factors such as TNF-α, IL-1β and IL-6 (Hayden and Ghosh, 2011). Some drugs reduce neuroinflammatory responses by inhibiting MAPK and NF-κB pathways, so as to play a therapeutic role in brain disorders (Qin et al., 2018). Our study showed that both ATX and ASDZL treatment inhibited phosphorylation of MAPK and NF-κB signaling pathways. Therefore, our findings indicated that ASDZL reduced neuroinflammation in SHRs by repairing the BBB and inhibiting the MAPK and NF-κB signaling.

Our previous studies revealed that ASDZL improved NE and DA deficiency in the PFC and striatum of SHRs (Lei et al., 2017; Song et al., 2018). Besides, the expression of DA receptor (DRD)-1, DRD2, DRD3, DRD4, DRD5 and alpha2A adrenergic receptor in SHRs were up-regulated by ASDZL (Liu et al., 2011; Zhang et al., 2013; Lei et al., 2015). In addition, ASDZL affected synthesis, transfer and release of DA and NE to achieve the aim of regulation the brain DA and NE content (Zhou et al., 2017a; Zhou et al., 2017c; Zhou et al., 2018; Song et al., 2020a; Lei et al., 2020). Moreover, our studies revealed that ASDZL regulated AC/cAMP/PKA, CDK5/DARPP32/PP1 and Akt/GSK*-*3β/β*-*catenin pathways to play a role in DA signaling in postsynaptic membrane (Sun et al., 2016a; Sun et al., 2016b; Sun et al., 2017b; Zhou et al., 2017b; Sun et al., 2018). It can be noticed that our previous studies mainly focused on dopaminergic system in SHRs, which is known to be sensitive to inflammation during development (Baharnoori et al., 2013; Luchicchi et al., 2016). A study on chronic social stress found that diminished cAMP synthesis damaged BBB integrity, thus inducing neuroinflammation (Zhang et al., 2020). However, the specific mechanism of DA system and neuroinflammation in the pathogenesis of ADHD has not been studied. Further research is needed to seek pharmacological mechanisms of ASDZL on interaction between inflammatory mechanism and DA deficiency theory in ADHD.

Collectively, our work provided a preliminary explanation of the neuroinflammatory mechanism of ADHD with activation of immune cells in the brain, BBB injury and activation of MAPK and NF-κB signaling. Moreover, we demonstrated that the traditional Chinese medicine prescription ASDZL attenuated the core symptoms of ADHD via anti-inflammatory effects.

## Data Availability Statement

The original contributions presented in the study are included in the article/Supplementary Material, further inquiries can be directed to the corresponding authors.

## Ethics Statement

The animal study was reviewed and approved by The Animal Ethics Committee of Nanjing University of Chinese Medicine (No. 201910A041).

## Author Contributions

YS and XH contributed to conception and design of study. HY, TC, and ML contributed to acquisition of data. TC and SL contributed to analysis of data. YS and HY contributed to drafting the manuscript. SL and ML contributed to revising the manuscript critically for important intellectual content. All authors approved the version of the manuscript to be published.

## Funding

This work was financially supported by National Natural Science Foundation of China (Nos. 81674023, 81873342, and 82004416), and Postgraduate Research and Practice Innovation Program of Jiangsu Province, China (Grant No. KYCX20_1466). Project of Natural Science Research of the Higher Education Institutions of Jiangsu Province, China (No. 20KJB360001).

## Conflict of Interest

The authors declare that the research was conducted in the absence of any commercial or financial relationships that could be construed as a potential conflict of interest.
